# Progressive 24-Hour Recall: Usability Study of Short Retention Intervals in Web-Based Dietary Assessment Surveys

**DOI:** 10.2196/13266

**Published:** 2020-02-03

**Authors:** Timur Osadchiy, Ivan Poliakov, Patrick Olivier, Maisie Rowland, Emma Foster

**Affiliations:** 1 Open Lab School of Computing Newcastle University Newcastle upon Tyne United Kingdom; 2 Centre of Organisational and Social Informatics Faculty of Information Technology Monash University Melbourne Australia; 3 Human Nutrition Research Centre Newcastle University Newcastle upon Tyne United Kingdom

**Keywords:** computer systems, nutrition surveys, diet records, nutrition assessment, epidemiologic methods

## Abstract

**Background:**

Under-reporting because of the limitations of human memory is one of the key challenges in dietary assessment surveys that use the multiple-pass 24-hour recall. Research indicates that shortening a retention interval (ie, the time between the eating event and recall) reduces the burden on memory and may increase the accuracy of the assessment.

**Objective:**

This study aimed to explore the accuracy and acceptability of Web-based dietary assessment surveys based on a progressive recall, where a respondent is asked to record multiple recalls throughout a 24-hour period using the multiple-pass protocol and portion size estimation methods of the 24-hour recall.

**Methods:**

The experiment was conducted with a dietary assessment system, Intake24, that typically implements the multiple-pass 24-hour recall method where respondents record all meals they had for the previous day on a single occasion. We modified the system to allow respondents to add multiple recalls throughout the day using the multiple-pass protocol and portion size estimation methods of the 24-hour recall (progressive recall). We conducted a dietary assessment survey with 33 participants, where they were asked to record dietary intake using both 24-hour and progressive recall methods for weekdays only. We compared mean retention intervals (ie, the time between eating event and recall) for the 2 methods. To examine accuracy, we compared mean energy estimates and the mean number of reported foods. Of these participants, 23 were interviewed to examine the acceptability of the progressive recall.

**Results:**

Retention intervals were found to be, on average, 15.2 hours (SD 7.8) shorter during progressive recalls than those during 24-hour recalls. We found that the mean number of foods reported for evening meals for progressive recalls (5.2 foods) was significantly higher (*P*=.001) than that for 24-hour recalls (4.2 foods). The number of foods and the amount of energy reported for other meals remained similar across the 2 methods. In interviews, 65% (15/23) of participants said that the 24-hour recall is more convenient in terms of fitting in with their daily lifestyles, and 65% (15/23) of respondents indicated that they remembered meal content and portion sizes better with the progressive recall.

**Conclusions:**

The analysis of interviews and data from our study indicate that progressive recalls provide minor improvements to the accuracy of dietary assessment in Intake24. Additional work is needed to improve the acceptability of progressive recalls in this system.

## Introduction

### Background

There are different methods for assessing dietary intake of a population by either measuring markers of nutrient intake (eg, doubly labeled water for measuring energy expenditure) or surveying the intake of foods and drinks (eg, food frequency questionnaires and 24-hour recalls) [1,2]. A successful method is expected not only to be cost-effective and scalable and to estimate dietary intake with acceptable accuracy but also to impose a low subject burden to reduce the likelihood of participant attrition and misreporting because of reactivity bias (ie, changes in respondents’ eating behavior in response to the act of recording) [3-8]. One of the most widely adopted approaches is the multiple-pass 24-hour recall, which is considered to offer a favorable balance of those characteristics [9]. However, in a validation with adults aged 20 to 60 years, Lopes et al [10] found the interviewer-led multiple-pass 24-hour recall method to underestimate habitual energy intake by 33% compared with energy expenditure measured using the gold standard method, doubly labeled water. The estimation error may, in part, be associated with recall bias because the accuracy of the 24-hour recall method relies on respondents being able to retain details about intake for a relatively long period [1,3,11,12].

According to Macdiarmid and Blundell [3], recalling intake even for the previous day is a challenging task for some individuals. Dietary assessment is especially difficult with certain population groups, for example, with people with reduced cognitive and memory abilities (eg, fading memory and reduced attention span) [13]. Human memory and lack of attention introduce such errors as unintentional food omissions, which can contribute significantly to underreporting of dietary intake. Memory errors may also reduce the accuracy of a method used for portion size self-estimation, for example, photographs of various food serving sizes presented to respondents [14-16]. The serving size that a respondent remembers that they ate, the portion size consumed in reality, and the portion size presented in the photograph may be different [17-19]. In addition, misreporting may occur when respondents are asked about specific details of recipes used for cooking of the reported foods [17]. Especially, if the meal was not cooked by the respondent, they can easily misreport its ingredients [17].

The emergence of dietary assessment systems that automate the 24-hour recall method offers a multitude of benefits, including cost-efficiency and scalability [15,20-22]. Individual interviews in such a system are replaced with a Web-based survey, where thousands of respondents can record and submit their dietary recalls remotely. However, Web-based dietary assessment surveys mostly implement an interviewer-led multiple-pass 24-hour recall procedure. With some of its methodological elements, these systems inherit its limitations, including errors related to human memory [1,16]. Specifically, these systems inherited a long-time interval between eating event and recall. For example, respondent will likely report breakfast at least 24 hours after its consumption with the 24-hour recall. Meanwhile, the self-administered manner of Web-based surveys allows exploring the use of shorter retention intervals that could potentially improve the accuracy of dietary assessment [23,24].

The multiple-pass 24-hour recall method was designed specifically to reduce misreporting in self-estimated intake because of errors related to human memory and attention [25,26]. However, evaluations show that underreporting and omissions of intake in 24-hour recalls are still common occurrences [16,27]. Memories of eating and drinking start deteriorating even an hour after a meal [28,29]. Indeed, research by Baxter et al [23,24] indicates that shortening the retention interval may increase the accuracy of a dietary intake recall. In 2 studies, children were observed eating 2 school-provided meals and interviewed to obtain a 24-hour recall. In the first study, children were interviewed using 1 of 6 interview conditions achieved by crossing 2 target periods (prior 24 hours and the previous day) with 3 interview times (morning, afternoon, and evening) [23]. In the second study, the interviews were conducted either the same day in the afternoon (shorter retention interval) or in the morning for the previous day (longer retention interval) [24]. In both cases, the correspondence rates for the observed/reported energy and the number of reported food items were higher when interviews were conducted after a short period. The first study revealed that the highest correspondence rate for energy and macronutrient intake occurred for the interviews conducted in the afternoon and in the evening for the immediate prior 24-hour intake period and the lowest for previous day recalls (midnight to midnight) conducted in the afternoon and in the evening [23]. Participants of this study were children, and a positive effect of short retention interval for the accuracy of the 24-hour recall is yet to be demonstrated with other population groups. At the same time, the benefits of short retention intervals can be seen in other dietary assessment methods. The weighed food diary method that asks respondents to record all foods and drinks at the time of consumption and has theoretically shorter recall interval is considered to be less prone to memory errors [11]. However, this method has the potential disadvantage of reactivity bias in intake reports and even changing respondents’ diets because of the burden of weighing and recording [3-8]. To collect accurate records, this method assumes subjects have access to scales at the time of preparing their food and are able to use them competently [14,30].

### Objective

This research proposes a progressive recall method, where a respondent is asked to record multiple recalls of meals throughout the day. Contrary to the weighed food diary method, the progressive recall uses the multiple-pass procedure and portion size estimation methods of the 24-hour recall method. The progressive recall does not require recording intake at the time of consumption and uses food photographs for portions size estimation instead of weighing foods and drinks using scales. The progressive recall theoretically requires respondents to remember less information over short periods, which reduces the burden on their memory and potentially increases the accuracy of dietary assessment. The a priori hypothesis of this research is that the respondent would report more foods and energy per a single recall and per an individual meal during a progressive recall. This study provides an overview of Intake24 designed for conducting large-scale dietary surveys based on the multiple-pass 24-hour recall method; and modifications added to the system to enable the progressive recall method. The study then describes the design and reports the results of a study that compared 24-hour and progressive recall methods in Intake24. This study examines the effects of using the progressive recall on the accuracy and acceptability of the system.

## Methods

### Intake24

Intake24 is an open-source system developed at Newcastle University to administer large-scale dietary surveys. The system automates a multiple-pass 24-hour recall method [16]. Intake24 was validated against interviewer-led recalls, with 180 participants aged 11 to 24 years [16]. The system has been field tested in those aged from 11 years to older adults to examine the feasibility of using Intake24 with the Scottish population on a large scale [31]. Both studies found Intake24 to be of comparable accuracy to the interviewer-led 24-hour recall method. The accuracy of energy intake estimated by the system was validated using doubly labeled water [32].

**Figure 1:**
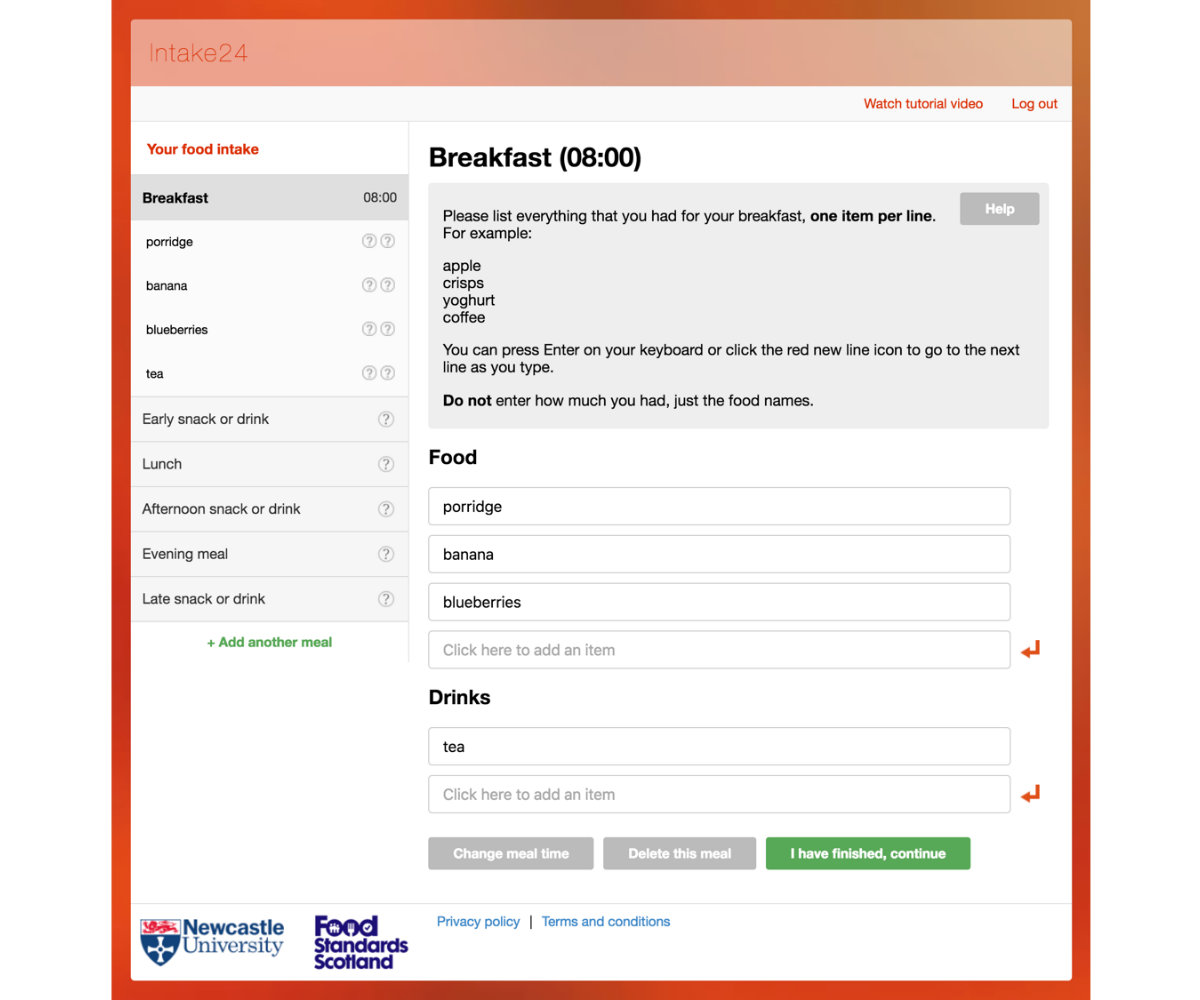
List of meals, food, and drinks names in a free text format in Intake24.

**Figure 2:**
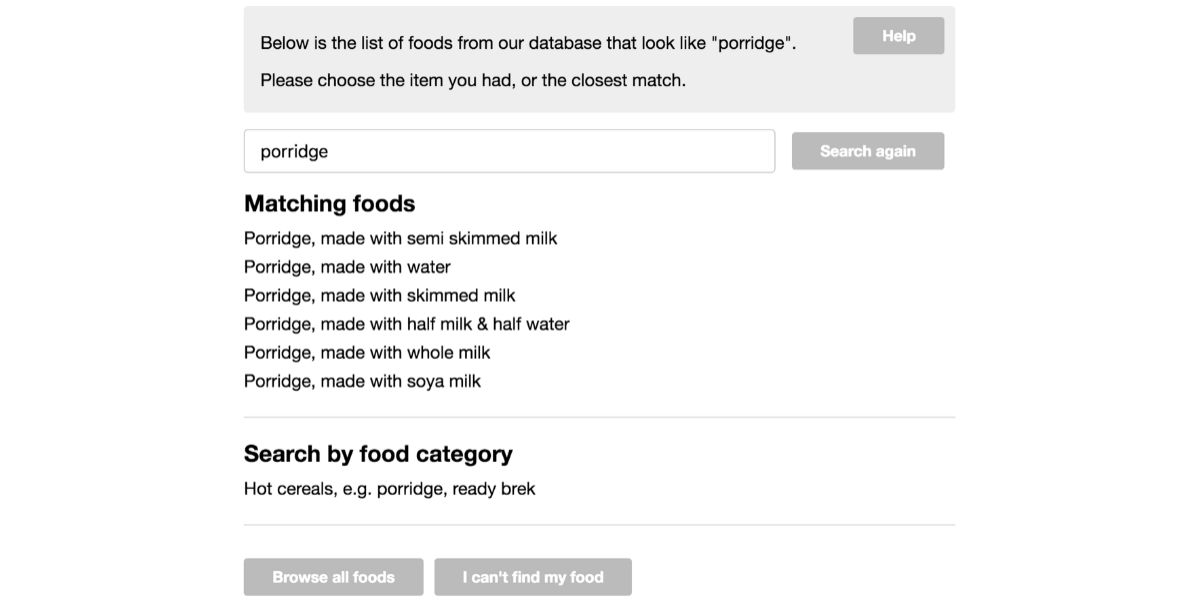
Search results returned in response to a food name typed in a free text format in Intake24.

**Figure 3:**
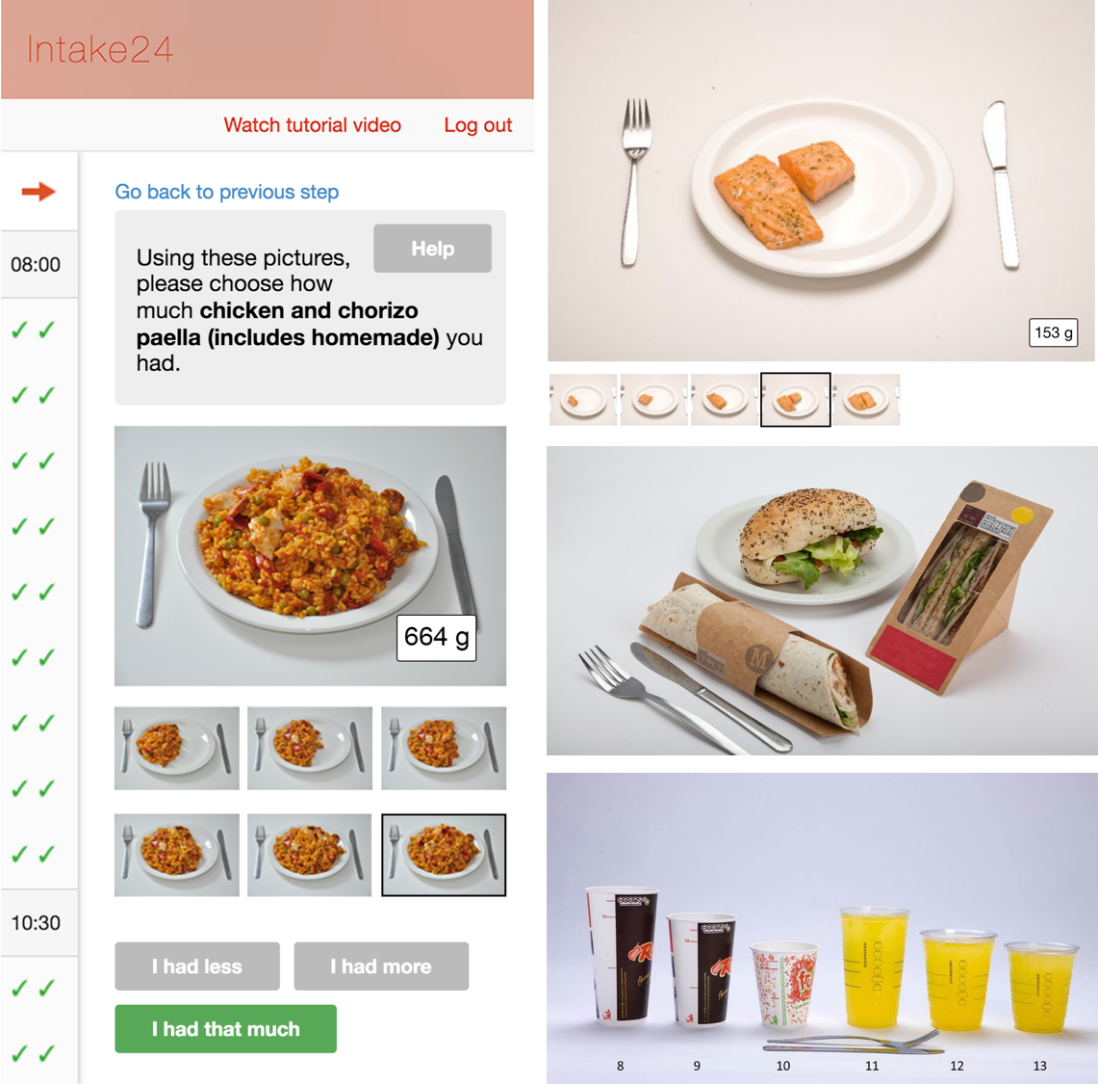
Food serving size estimation with photographs used in Intake24.

Typically, respondents in Intake24 perform a recall in the morning on 3 or 4 nonconsecutive days to capture a wide variety of foods eaten. Respondents are asked to answer a series of questions about meals they consumed for the previous day in a Web-based survey. The survey interface is optimized for desktop and mobile devices [20]. The structure of the survey generally follows the questionnaire of the multiple-pass 24-hour recall method with some deviations. In the first pass, respondents are asked to recall all meals they had for a previous day (Figure 1). Respondents select the name of a meal from a list of suggestions (breakfast, lunch, evening meal, and early/afternoon/late snack or drink) or they can type a new name for the meal. In this pass, for every meal, respondents are also asked to provide the list of foods and drinks in a free text format. In the second pass, for every name of a food or a drink typed in a free text format, respondents search and select specific records from a taxonomy of around 4800 foods (Figure 2). As the method of portion size estimation, Intake24 uses validated photographs of weighed servings. In this pass, for every reported food and drink, respondents are also asked to select a photograph that most closely resembles the serving size they had (Figure 3). In the third pass, respondents review the list of reported meals, foods, and drinks and submit their recall. A single submission typically includes 4 to 7 eating occasions (eg, breakfast, morning snack, and lunch). At the end of a study, Intake24 produces a report for researchers that contains an estimated portion size, energy, and nutrient intake for each reported food and drink. Energy and intakes of macro- and micronutrients are calculated using the national food composition tables from the region where the population was surveyed where possible.

Before taking part in a study, respondents are asked to specify the time in the morning (before 10 am) for recording their meals for a previous day using the 24-hour recall method. On recall days at the specified morning time, participants receive automated reminders to submit their intake for the previous day in the form of text messages on their mobile phones and via emails. Respondents access the survey in Intake24 using a secure personal URL that is included in the text of the reminder. The reminder contains the following text: “*Morning {Person’s Name}. It's time to record your diet for YESTERDAY. Follow this url to login: {PERSONAL URL TO THE SURVEY}*.”

### Progressive Recall

**Figure 4:**
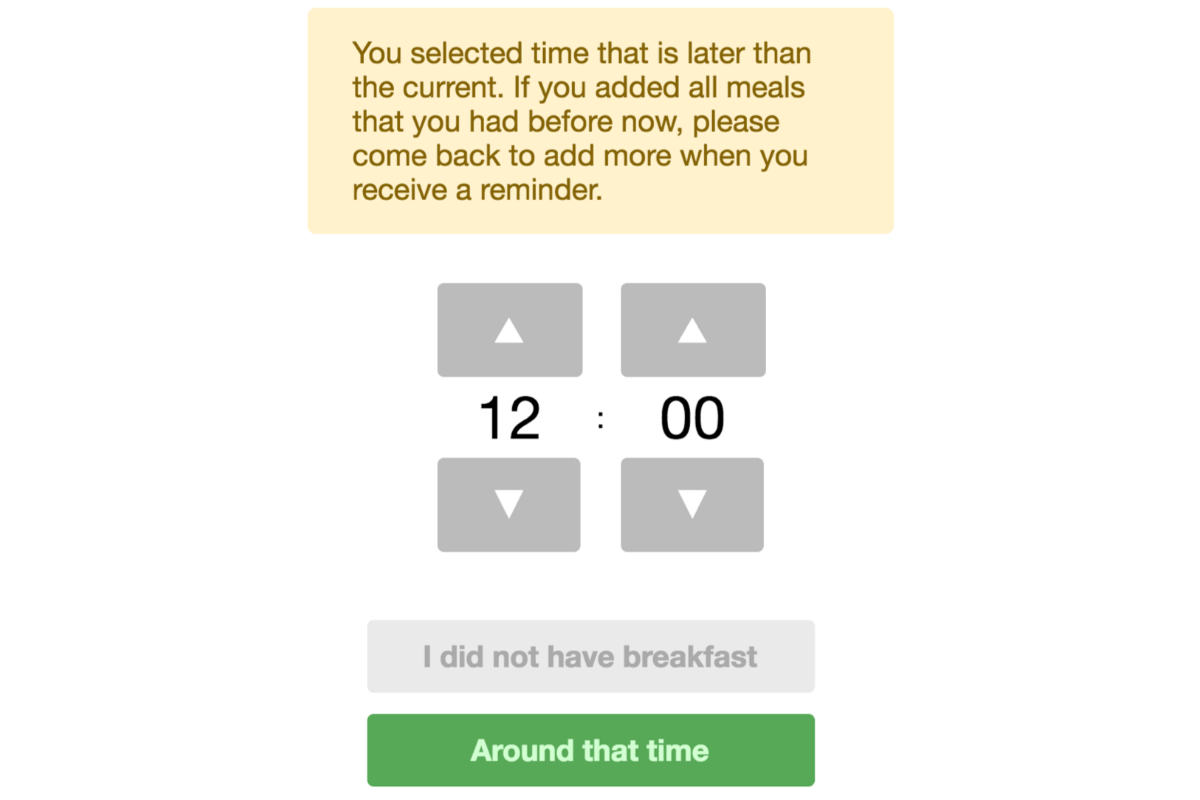
Warning message in Intake24 when a user tries to log meals before the actual intake.

To explore the potential of improving the accuracy of dietary assessment results produced by Intake24 by reducing the retention interval (ie, time between an intake and a recall), this research implemented a modified version of the system that allows recording intake as the day progresses. Although using the same multiple-pass procedure and portion size estimation methods with photographs of serving sizes of the multiple-pass 24-hour recall described in the section Intake24, progressive recalls ask respondents to make at least three submissions on the day of a survey and 1 submission the next morning. In the first 3 submissions, subjects report morning, afternoon, and evening meals. On the next morning, they report late meals or snacks for the previous day. For example, in the first submission of the progressive recall, respondents typically report only their breakfast and morning snacks using the multiple-pass procedure. If participants select a time of meal that is later than the current time, the system alerts the respondent and does not allow submission of that meal (Figure 4).

When respondents register to take part in a study, they are asked to provide 3 time points to record meals for the same day to personalize their reminders for progressive recalls. These time points are expected to fit their usual eating patterns and daily plans. The first time point before 12 pm, for recording breakfast, morning snacks, and drinks; the second between 12 pm and 4 pm, for lunch, afternoon snacks, and drinks; and the third after 4 pm, for dinner, evening snacks, and drinks. Respondents are additionally asked to provide a time point on the next morning before 10 am to record late meals and snacks and finalize their recall for the previous day. On the days of progressive recalls, participants receive 3 reminders at the times specified by them to add meals into the system as the day progresses in the form of text messages on their mobile phones and via emails. As in 24-hour recalls, respondents access the survey using a secure personal URL that is included in the text of the reminder. The reminder to submit morning intake contains the following text: “*Morning {Person’s Name}. Today you should record your diet for TODAY as the day goes on. Follow this url to login: {PERSONAL URL TO THE SURVEY}*.” The reminder to submit afternoon and evening intake contains the following text: “*Hi {Person’s Name}. It's time to continue recording your diet. Follow this url to login: {PERSONAL URL TO THE SURVEY}*.” Finally, the reminder to complete their recall the next morning contains the following text: “*Morning {Person’s Name}. Please don’t forget to submit your dietary recall that you started yesterday. Follow this url to login: {PERSONAL URL TO THE SURVEY}*.”

### Recruitment

To investigate the effectiveness of using the progressive recall in automated dietary assessment systems, we conducted a dietary survey, where we compared the 24-hour recall with the new method. Before data gathering, Newcastle University Ethics Committee granted the ethical approval for the study (reference number: 4971/2018). We recruited participants for the survey by circulating an advertisement with a detailed description of the study and a link to a registration Web form via the internal email system of Newcastle University. The first page of the Web form contained more details about the study as well as a consent form. Participants could only proceed to registration for the study once they accepted all clauses of the consent form. To take part in the study, candidates had to be 18 years or older, speak English, have a diet that is considered common for the United Kingdom, and agree not to change their diet during the study. For completing 6 dietary recalls, participants were offered a £30 Amazon voucher. The aim of this study was to support our hypothesis with a view to validate it on a larger scale if the results indicate the benefits of the progressive method. For that reason, we did not pose any requirements to the demographics of our respondents. However, we aimed to have a gender-balanced recruitment. We recruited 50 participants (26 males and 24 females) with an age range between 18 and 64 years.

### Procedure

Participants were asked to complete their recalls on 2 consecutive weeks. During each week, we asked participants to log in to Intake24 and complete 3 dietary recalls on 3 consecutive days between Monday and Friday. We used a cross-over design and surveyed participants using the 24-hour recall during 1 week and the progressive method during another. The study resulted in 35 participants recording their intake using the 24-hour recall method on the first week and using the progressive recall method on the following week. The remaining 15 participants used the 2 methods in the reverse order. To have a balanced sample size in each group, we randomly excluded half of the participants from the first group. Thus, for the analysis, we used recalls from 33 participants (15 females and 18 males). The first of each type of recall was used to minimize the learning effect by familiarizing participants with the interface of the system and the procedure. For that reason, the first day of each type of recall was excluded from analysis, leaving 4 days of recalls from every individual. Participants were asked to avoid changes in their diets and not to record their meals elsewhere (eg, notepads) to aid their recalls.

We did not ask respondents to record their intake on the weekends, as it is normally recommended for conducting dietary assessment studies using the multiple-pass 24-hour recall [9]. Respondents were informed about the schedule of their recalls 2 days before the first one, which could affect their diets. However, the primary goal of this research was finding deviations in estimated dietary intake with the 2 methods implemented within the same system Intake24. For that reason, we assume that the limitations of the study design affect the accuracy of both types of recall. Thus, if there is a difference in the accuracy of estimated dietary intake with the 2 methods, it still can be observed.

### User Interviews

To analyze the usability and acceptability of the progressive recall, we offered participants to share their experience of the 2 methods in an interview after their last recall. Interviews were arranged with 23 participants (P1 to P23; 18 males and 5 females) aged between 18 and 44 years. The interviewer asked respondents *which type of recall, if any, was more convenient for them and which type of recall, if any, helped them to remember foods better*. Respondents were asked to elaborate on these 2 topics. The interviews were audio recorded and transcribed. The transcripts were thematically analyzed, and this paper discusses the topics that emerged during the analysis [33].

### Statistical Analysis

We compared the mean retention intervals for meals reported during progressive recalls with those reported during 24-hour recalls. The analysis provides information about the mean number of times respondents logged in to system during a single progressive recall and the type of devices (eg, desktop and mobile) used by respondents during this study. We also compared the mean number of foods and energy reported for a single day and for individual meals reported using the 2 methods. Meals reported with 1 type of recall that did not have a pair reported by the same respondent in the other type of recall were excluded from the analysis. For example, if the respondent reported breakfast during a 24-hour recall but did not report it during a progressive recall, that meal was excluded. If they reported breakfast twice during 24-hour recall but did it only once during progressive recalls, then 1 meal was excluded from the 24-hour recall. Thus, for each user, we compare the same number of recalls and the same number of meals across the 2 methods of recall. Food items that can be reported by respondents include drinks and condiments (eg, pear juice, ketchup, and sour cream in soup). Ingredients of a salad or a sandwich are considered as separate foods. Each food item can be reported more than once for a single day and for a single eating occasion (meal). Validations of Intake24 demonstrate that as with interviewer-led 24-hour recalls, food omissions commonly occur in recalls collected using the system [16,31]. For that reason, the analysis assumes that an increase in the number of reported foods is a likely indication of an increase in accuracy of the method. The significance of difference between the means is analyzed using dependent *t* test for paired samples. The analysis uses histograms to visualize that difference. A larger number of foods in a meal may potentially make it harder to remember. In addition, meals that contain the same foods day to day may potentially be easier to remember for respondents. For these reasons, we examine the mean size of each meal and the mean number of distinct foods reported over the study by a single respondent in each meal.

## Results

### User Interviews

In the interviews, exploring participants’ experiences of the 2 different types of recall, 65% (15/23) participants stated that they preferred the 24-hour recall method, 30% (7/23) preferred the progressive method, and 4% (1/23) remained neutral. The major advantage of the 24-hour recall described by respondents was them being able to record meals on a single occasion without, as 1 participant said, *“changing my life routine too much”* (P2). Despite notifications being sent at the times customized for each respondent, these often did not fit into their actual daily plans, for example, participant (P10) said:

If I’m really busy in a day and I’ve not really had a break between breakfast and lunch, I won’t necessarily get a chance to record what I had for breakfast until like 2 o’clock.

They then added that being able to change previously defined notification preferences would help to address that issue:

I think you should give an option for changing the times of the prompts... I set down time for my breakfast and then I realized that the prompt that I was getting was actually when I was travelling to work.

Three respondents (P2, P9, and P14) stated that doing their recalls in the evenings was especially difficult for them. For example, respondent (P14) said:

I find it really difficult to do any work at night... Usually you have food, you have dessert, then you’re in relaxation mode. So, to bring yourself to do work is really difficult at like 10:00-10:30 p.m. You’re getting ready for bed... So the last thing you want to do is do a study form.

In contrast, however, another 3 respondents (P6, P12, and P19) suggested replacing the morning recall with an evening recall after the last meal in the 24-hour recall method.

Despite these difficulties, of all interviewed participants, 65% (15/23) stated that the progressive recall helped them to better remember the foods and drinks they had consumed. However, although participant (P1) stated that the 24-hour recall fit better into their lifestyle, they did experience the following issue with this recall method:

I think I must have eaten something cause I didn’t have lunch until like two o’clock. But I don’t really remember. I was actually guessing today. I was guessing about yesterday.

Respondents who expressed their favor toward the progressive method said that short retention intervals assisted them recalling more details about their meals. For example, participant (P18) noticed that she remembered serving sizes better during progressive recalls:

I think the portion size in general was hard especially with foods like where there were multiple components and they were all mixed together. So, how do you remember exactly how much something was? So, I think I was more accurate when I did it after every meal.

Respondent (P17) also pointed out that memorizing foods is not a casual task, and for that reason, recording their diet as the day progressed worked better for him:

The previous day was a bit of a task because I couldn’t remember the small details and I relied more on the Intake24 to actually remind me like butter and bread... The small thing I would forget. Looking back for the previous day there was a lot of information that I tried to hold considering it’s not something that you normally commit to memory. However, I’ve really actually enjoyed this week just going through it [diet] as the day progresses.

Some respondents stated that short retention intervals were helpful in recalling irregular eating patterns. For example, this is how (P14) compared the 2 types of recall:

The second one [24-hour] obviously relies on a lot more memory, which is difficult, especially when you had days when you’ve eaten out and you had a few different types of snacks... The days, I had consistent meals, my regular lunch and dinner, it was really easy next day because I have three coffees and ... the same soup, but then ... I ate a Lebanese food one evening and I had food outside during the afternoon as well and the next day I was like, “Ah, so many different ingredients to remember!”

This experience is supported by another respondent (P12):

One day when the school had put on like a buffet, and I had some things from the buffet, and the next morning I couldn’t remember exactly what I had. So, yeah, I think it’s definitely easier to remember in the moment.

### Statistical Analysis

The study resulted in 63 submissions for each type of recall. Respondents, on average, logged in to the system to report their meals 3.0 (SD 1.6) times per day during progressive recalls. Retention intervals were found to be, on average, 15.2 (SD 7.8) hours shorter during progressive recalls than those during 24-hour recalls. During the week when respondents were surveyed using the 24-hour recall method, 46 and 17 submissions were made from desktop and mobile devices, respectively. In this period, 5 respondents switched between desktop and mobile device between recalls. During the week of progressive recalls, 42 and 35 submissions were made from desktop and mobile devices, respectively. In this period, 10 respondents switched between desktop and mobile device during a single progressive recall. No tablet devices were recorded to be used by respondents during this study.

The mean number of foods recorded for a single day was not significantly different for the 2 methods (*P*=.12). In the 24-hour and progressive recall methods, respondents, on average, reported 12.7 and 13.9 foods, respectively, per a single submission. The mean energy reported with the 2 methods also remained similar (*P*=.18) with 1668.9 kcal and 1529.7 kcal for the 24-hour and progressive types of recall, respectively. The same trend remained across all individual meals except for the evening meal (Table 1). The mean number of foods reported for evening meals during progressive recalls (5.2 foods) was significantly higher (*P*=.005) than during 24-hour recalls (4.2 foods).

As can be seen from Table 2, evening meals had the largest number of distinct foods reported over the study by a single respondent (ie, mean variety). At the same time, evening meals had the largest mean number of reported foods per a single submission. In other words, evening meals were the largest in size, but foods in those meals were the least repetitive. This could make them harder to remember and could explain the significant difference in the number of reported foods with the 2 methods observed only for evening meals.

**Table 1:** Size and energy contents of meals reported with conventional and progressive 24-hour recall methods.

Meal	Number of foods, mean (SD)				Energy (kcal), mean (SD)		
	24 hours	Progressive	*P* value	24 hours		Progressive	*P* value
Afternoon snack or drink	2.7 (1.2)	2.7 (1.5)	.43	325.0 (433.1)		217.9 (263.4)	.30
Breakfast	3.6 (1.6)	3.8 (1.7)	.51	373.5 (383.9)		318.2 (205.0)	.41
Early snack or drink	2.6 (1.7)	2.1 (1.1)	.19	120.8 (145.3)		126.1 (192.0)	.87
Evening meal	4.2 (1.9)	5.2 (1.8)	.005	655.3 (378.0)		732.8 (404.3)	.32
Late snack or drink	2.3 (1.0)	3.0 (2.0)	.31	372.4 (426.2)		318.4 (362.5)	.74
Lunch	3.9 (1.8)	4.0 (1.8)	.68	592.6 (349.1)		491.3 (255.3)	.09
Late snack or drink	2.3 (1.0)	3.0 (2.0)	.31	372.4 (426.2)		318.4 (362.5)	.74
Full day	12.7 (5.5)	13.9 (6.1)	.12	1668.9 (851.3)		1529.7 (834.7)	.18

**Table 2:** Mean varieties and sizes of meals reported during the study.

Meal	Variety, mean (SD)	Size, mean (SD)
Evening meal	12.9 (4.8)	4.7 (1.9)
Lunch	11.5 (4.3)	3.9 (1.9)
Breakfast	7.8 (2.9)	3.7 (1.7)
Afternoon snack or drink	5.7 (2.4)	2.5 (1.4)
Early snack or drink	4.3 (2.8)	2.3 (1.5)
Late snack or drink	5.5 (4.3)	3.9 (1.9)

## Discussion

### Principal Findings

More than half of the respondents in our study preferred the 24-hour recall method for the previous day because it was easier to integrate into their daily routine. At the same time, from our interviews, we found that in many cases, respondents did not have time to complete a recall when they received a reminder. The reminders were customized by the administrators at the beginning of the study to fit a normal eating pattern of each respondent. However, the actual timing of eating events for some respondents was different during the study. For other respondents, notifications did not account for their plans for those days and distracted them. These factors could cause negative reaction to the progressive recall captured in our interviews. Thus, giving respondents the ability to change their notification preferences in the survey interface of Intake24, for example, postpone the received reminders, could potentially improve the acceptability of the progressive recall method. Another potential option is to give respondents the ability to decide the number of recalls they want to make during the day. That could help to identify a comfortable number of recalls that help memory of respondents without being intrusive.

Future research could potentially find improvements to the acceptability of progressive recalls in Intake24 and similar dietary assessment systems by examining user experience implemented in popular mobile apps for personal dietary assessment (eg, MyFitnessPal and Lose It!) [34]. Such apps allow respondents recording their intake progressively. An audience of millions of users voluntarily tracking their diet on a daily basis demonstrates a certain level of acceptability of the progressive method used in these dietary apps. At the same time, recording intake in a mobile dietary app is comparable in terms of tasks and difficulty with that in a dietary survey. Thus, the user experience of mobile dietary apps could be used as a source of inspiration for addressing acceptability issues identified in this research.

The statistical analysis of data collected in this study shows that retention intervals for meals reported during progressive recalls are significantly shorter compared with those for meals reported during 24-hour recalls. A significant difference in the number of foods reported with the 2 methods was observed for evening meals only, where respondents reported more foods during progressive recalls. The size and energy content of other meals and the overall daily intake remained comparable with that reported during the 24-hour recalls. A larger variety of foods in evening meals that were identified during analysis could make this type of meal harder to recall the next morning but easier shortly after consumption. Furthermore, irregular eating patterns were suggested to be difficult to remember by some participants in our interviews. In contrast with our study design, 24-hour recall surveys often include longer time gaps between recall days and a mixture of week and weekend days, aiming to capture more variety in individual dietary intake [16]. Such variety is likely to increase the burden on human memory, and it is possible we would observe the advantages of the progressive recall for other meals and snacks in studies conducted over long periods. That is supported by those participants in our study who suggested that shorter retention intervals helped them to remember more details about their intake such as portion sizes.

### Limitations

This study involved a relatively small number of participants and did not use any method of randomization of participants. The recruitment method meant that the demographics of our respondents were limited, which may mean that the results do not generalize to a wider population. Only a subset of participants from the recruited sample agreed to take part in our interviews. Owing to the study design, we are comparing 1 day’s intake against intake from another day, and therefore, it is impossible to determine whether the observed difference is because of the method or to day-to-day variation in intake. In addition, we did not collect intake records for weekend days. This limits the generalizability of the findings to weekdays only. For a more reliable judgment of the accuracy of energy intakes estimated with the progressive recall, they could be compared against true intake measured by direct meal observation or using objective biomarkers of dietary intake.

### Conclusions

In this paper, we aimed to address one of the key challenges in dietary assessment, which is unintentional underreporting because of poor human memory [3]. Previous research has demonstrated that the burden on memory can be minimized by reducing the amount of information that needs to be remembered along with the period it needs to be retained [23,24]. We proposed a modified procedure of the 24-hour recall that we refer to as a progressive recall. The modified method instead of requiring respondents to report their intake for the prior 24 hours or a previous day on a single occasion offers recording meals progressively, shortly after intake, while using the multiple-pass approach and portion size estimation methods of the 24-hour recall. The progressive recall was implemented in Intake24, a system for conducting large-scale population dietary surveys. The method was compared with the multiple-pass 24-hour recall that is also implemented in Intake24. Retention intervals were found to be significantly shorter during progressive recalls than those during 24-hour recalls. This research did not find a significant difference in the numbers of foods or the amounts of energy reported during progressive and 24-hour recalls for a single day in Intake24. Progressive recalls were found to capture more foods for evening meals. More than half of the interviewed respondents in our study found fitting multiple intake recalls into their daily lifestyles to be difficult and preferred the 24-hour recall method. To address concerns raised by respondents, we proposed methods for improving the acceptability of progressive recalls in Intake24 that could be investigated in the future. At the same time, a similar number of respondents pointed out that they remembered their intake better with the progressive method.
